# Case Report: A male newborn with occipital horn syndrome

**DOI:** 10.12688/f1000research.154409.1

**Published:** 2024-08-01

**Authors:** Marian K. H. Georgeos, Engy M. Hanna

**Affiliations:** 1 NICU Specialist, MRCPCH, MSc, Neonatal Intensive Care Unit, Arab Contractors Medical Center, Nasr City, Cairo Governorate, Egypt; 2 Neonatal Intensive Care Unit, King Fahad Medical City, Riyadh, Saudi Arabia

**Keywords:** Occipital horn syndrome, ATP7A gene, cutis laxa, copper transport disorders, occipital exostosis, Menkes disease.

## Abstract

Occipital horn syndrome (OHS) is a rare genetic disease and copper transport disorder caused by a faulty ATP7A gene with multisystemic presentations, most originally related to musculoskeletal and connective tissue affections. In our case, a male neonate with OHS presented soon after birth with pathognomonic occipital exostosis, cutis laxa at the nape region, and widely opened skull sutures and fontanels. A skeletal survey showed occipital exostosis projecting from the line of insertion of the trapezius muscle and wide fontanels on skull X-ray films with no exostoses or deformities elsewhere. In addition to our case report being the second reported case for the condition detected early in the neonatal period, it also emphasizes the importance of investigating any sign thoroughly, as it may be an early alarming sign of a progressive disease that may affect the patient’s quality of life. In addition, it highlights the value of early diagnosis and multidisciplinary management of these patients.

## Introduction

Despite the fact that copper is an element needed only for our body in minute amounts, any imbalance in its homeostasis will affect nearly all of our body systems. The entity “Copper transport disorders” comprises a disparate number of diseases including occipital horn syndrome (OHS, OMIM#304150) formerly called Ehler Danlos syndrome type IX or X-linked cutis laxa, and initially mentioned in the medical literature in 1975, ^1^ beside classical Menkes disease (CMD), ATP7A-related distal motor neuropathy (DMN), and Wilson disease. The first three conditions result from a faulty ATP7A gene and lead to copper deficiency, while the latter is caused by ATP7B gene defects and leads to copper overload. The ATP7A gene is present on the long arm of the X-chromosome, band 13.3 (Xq13.3), extends for 140 kilobases, encodes a protein built up of 1500 amino acids, and weighs approximately 165 kDa. This gene is expressed in nearly all cell types, except hepatocytes. ^2^

Mutations in this gene lead to either the formation of a distorted form of transmembrane copper transporting P-type ATPase protein or the cessation of its synthesis. ATPase plays a crucial role in copper homeostasis. Being a part of the trans-Golgi network (TGN), it helps to bring copper inside the Golgi network to be incorporated with copper-dependent enzymes; however, in cases of increased intracellular copper, this protein is mobilized to the cell membrane to help discard the excess. Copper-dependent enzymes include lysyl oxidase (LOX), which catalyzes the initial step of collagen synthesis. It also comprises the dopamine-β hydroxylase enzyme, which plays a role in the neurological and autonomic systems and helps form norepinephrine out of dopamine. Furthermore, it includes cytochrome C oxidase, superoxide dismutase, ascorbic acid oxidase, and tyrosinase. ^3^

Accordingly, a significant ATPase deficiency gives rise to Menkes disease, characterized by severe neurodevelopmental delay, but occipital horn syndrome occurs in the presence of a considerable amount of functioning protein. Despite being genetically related to the previous two diseases, ATP7A distal motor neuropathy, the third member of the ATP7A gene-dependent disease family, does not exhibit any similar biochemical or clinical manifestations besides neurological effects in the form of motor neuron disease. ^4^

## Case report

Our case report describes a male full-term neonate born at 39 weeks of gestation by spontaneous vaginal delivery to non-consanguineous parents. The APGAR scores were 8 and 9 in 1 ^st^ and 5 ^th^ minutes, respectively. The mother was 25 years old primi gravida. She attended antenatal visits regularly, and nothing was relevant to her medical history. Antenatal scans showed mild ventriculomegaly in the fetal brain.

On admission, the baby’s vital signs were stable, aside from being tachypneic (RR, 68). On plotting the body measurements on the growth chart, the weight was 2950 g (17 ^th^ percentile), length was 50 cm (47 ^th^ percentile), and head circumference was 32.5 cm (6 ^th^ percentile).

**Figure 1.**
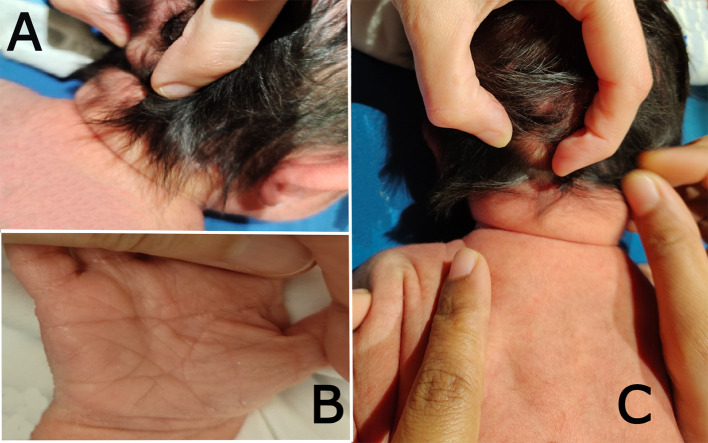
Clinical photographs of the patient: (a) shows the bony prominence projecting from the middle area of the occipital bone at the insertion site of the trapezius muscle. (b) the right hand of the patient shows a single transverse palmar (simian) crease. (c) shows lax redundant skin fold of the nape.

**Figure 2.**
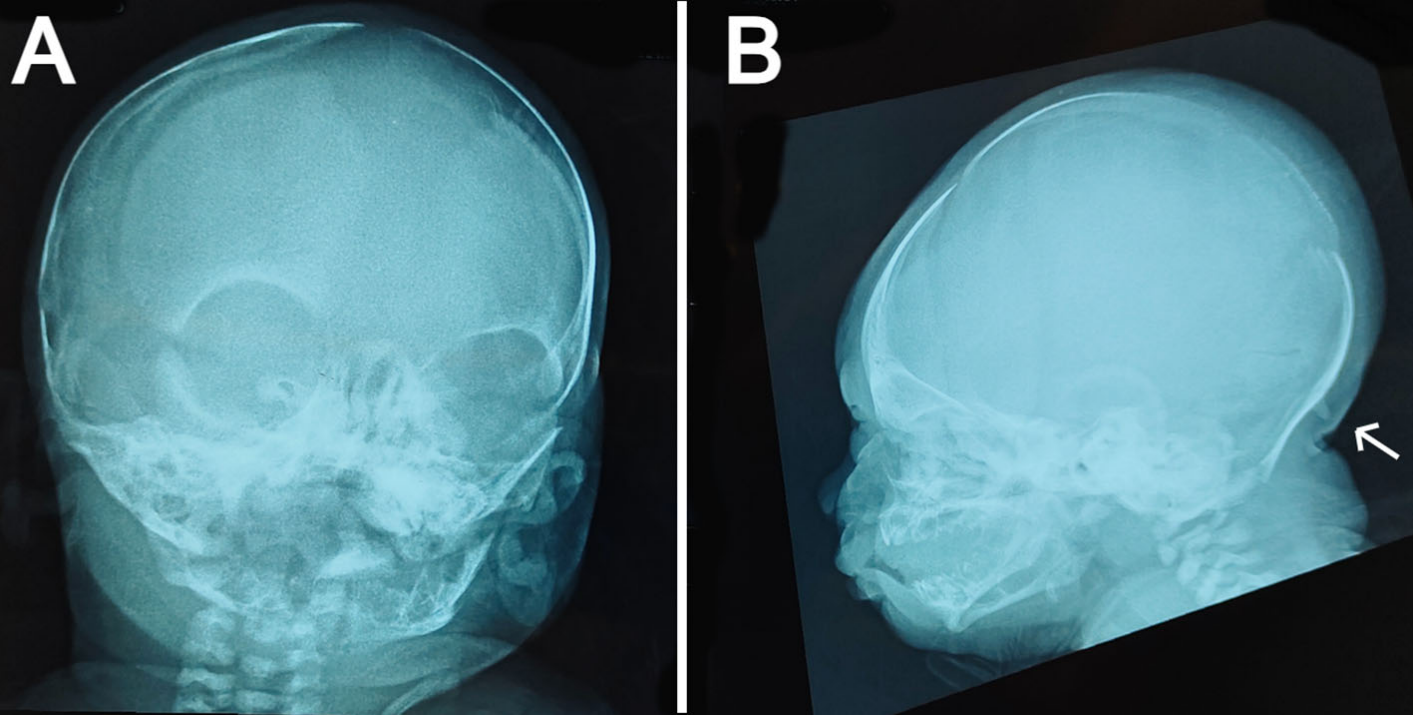
Skull x-ray films: (a) anteroposterior view showing widely opened anterior fontanel, sagittal suture, and metopic suture. (b) lateral view showing a bony prominence protruding from the occipital bone at the insertion site of the trapezius muscle (indicated by the white arrow) and redundant soft tissue at the nape.

On systematic head-to-toe review, multiple abnormal features were noted as follows: head inspection and palpation revealed bone protrusion projecting from the occipital area, roughly at the insertion of the trapezius muscle ( Figure 1A), a widely opened anterior fontanel, sagittal suture, and metopic suture, even though the head circumference was on the lower side of the normal. Additionally, complete ankyloglossia and lax redundant skin folds of the nape were observed ( Figure 2C).

On chest examination, the patient was tachypneic and could not maintain normal oxygen saturation without oxygen support. Regarding heart auscultation, normal heart sounds were heard in addition to a grade I diastolic murmur. Abdominal and limb assessments were normal, except for a single transverse palmar (simian) crease on the right hand ( Figure 1B).

The baby was admitted to the SCBU for investigation of the multiple special features mentioned before, and because of respiratory distress, he received oxygen via a nasal cannula. The cause of respiratory distress was speculated to be transient tachypnea of the newborn (TTN), as the baby was successfully weaned to room air in less than 24 h, and the sepsis work-up did not show any positive markers of infection. In addition, the baby developed neonatal jaundice on day one of life, which was mostly due to ABO incompatibility and received phototherapy. On day three of life, the baby was discharged, and the family was referred to a genetic center for counseling.

The anteroposterior view of the skull radiograph showed widely opened sagittal and metopic sutures and fontanels ( Figure 2A). Furthermore, a bony prominence protruding from the occipital bone was observed in the lateral view of the skull radiograph ( Figure 2B). Moreover, cranial ultrasound revealed cerebral asymmetry, with the right hemisphere being slightly larger than the left hemisphere. In addition, a tiny patent ductus arteriosus (PDA) and a small atrial septal defect (ASD) were reported by echocardiography. Routine laboratory blood tests including sepsis work-up and kidney and liver functions were performed, all of which were normal except for high indirect hyperbilirubinemia. No genetic tests were performed on the patient because of the lack of resources in our facility.

On follow-up visits at the pediatric outpatient clinic, the mother reported recurrent attacks of bronchiolitis over the baby’s first six months of life.

## Discussion

This is a noteworthy case report, as it is an extremely uncommon illness. To our knowledge, only 34 cases have been reported. Moreover, it is the first case diagnosed with occipital horn syndrome (OHS) in the Middle East, drawing the condition into the focus of healthcare personnel in this area and adding to the already known information worldwide.

Symptoms and signs of OHS are not readily apparent at an early age. This was concluded from the diagnosis age of the previously reported cases, as only one case, other than ours, was identified in the neonatal period. The hallmark observed in our patient was the occipital horn, a bony prominence projecting from the occiput at the insertion area of the trapezius muscle. This resembles what was mentioned by Beyens et al. in their review of cases in which almost all cases had this sign. ^3^ Fortunately, the mentality was mildly affected or unaffected at all, but this was very early to judge in our patient. In contrast to our patient, who had no obvious neurological problems, some reported cases exhibited hypotonia, developmental delay, and convulsions. In this context, most of the symptoms are related to connective tissue and musculoskeletal systems, which may progress to the extent of joint degeneration and movement difficulties. Regarding connective tissue manifestations, the majority of reported cases, including our case, presented with cutis laxa. Our patient had redundant skin folds in the nape region, which were suggested to be the remnants of the nuchal skin fold seen in antenatal ultrasound scans, although cutis laxa can occur in other places such as the face, chest, abdomen, or limbs. In agreement with Bonati et al., our baby had very wide sutures and fontanels. ^5^ This could be explained by the presence of abnormalities in the formation of collagen and elastin. Similarly, patients can present with coarse hair, joint hypermobility and dislocations, pes planus, inguinal hernias, and large cephalohematoma. As for skeletal problems, patients can have bone aches, kyphoscoliosis, thoracic cavity deformities, genu valgum, and long bone deformities, in addition to restrictive elbow movement and these signs seem to worsen by time. Fortunately, our baby had nothing of the previously mentioned skeletal problems and this might be due to the early age of diagnosis. In addition, urological manifestations are widely recognized in OHS, such as recurrent urinary tract infections, which may be due to bladder diverticula or hydronephrosis. Furthermore, dysautonomia problems frequently occur, such as hypothermia and orthostatic hypotension, which may be severe enough to cause syncope and chronic diarrhea, resulting in failure to thrive. Less commonly, patients can exhibit the following life-threatening conditions: intestinal obstruction, arrhythmia, and apnea attacks. ^6^^–^^8^ Moreover, our patient had signs that were not mentioned in any of the previously published cases as ankyloglossia and a single palmar crease.

On the other hand, most female members of the patients’ families are generally asymptomatic, apart from the pili torti reported in some cases. ^9^

A wide range of tests can be performed, including laboratory, imaging, molecular, and genetic tests. In terms of laboratory blood tests, patients with OHS are characterized by low serum levels of copper and ceruloplasmin. These tests are not very helpful in diagnosis at an early age because these levels are low by default in this period. ^9^ CSF examination revealed high catecholamine levels. ^7^ Fibroblast cultures exhibit increased intracellular copper. ^2^

Imaging can aid in the diagnosis and follow-up of patients. In our case, skeletal survey revealed occipital exostosis. Bone exostosis may also exist in long bones near the joints, which may limit joint movement. Furthermore, osteopenia, osteoporosis, fractures, bowing of long bones, widening of the metaphysis and diaphysis, flat vertebra, and short clavicles have been reported. ^3^ Pelvic and abdominal ultrasound should be performed annually to screen for bladder diverticula and hydronephrosis. ^9^ Assessment of lung function clinically and by spirometry plays an important role in these patients for many reasons: first, for the early detection of asthma. Second, these patients may have a higher risk of developing chest infections, as highlighted by our patient’s mother, over the first few months of our patient’s life, and may have a higher incidence of obstructive and restrictive lung diseases, as reported by studies conducted for other types of cutis laxa patients. ^10^ Cardiac investigations, including ECG, echocardiography, and MR angiography, are performed starting from puberty onwards, which may detect prolonged QT interval, mitral valve prolapse, and tortuous arteries such as hepatic, splenic, and cervical arteries, respectively. Predictably, echocardiography performed in our patient did not show any of these but only an ASD and a tiny PDA. Brain MRI may display cerebellar atrophy, delayed myelination, mild cortical atrophy, and convoluted intracranial arteries. ^3^ In the same context, cranial ultrasound showed asymmetrical cerebral hemispheres in our baby. This could be due to mild cortical atrophy, but we suggest that this should be confirmed using MRI.

Exploring the root of the disease, genetically, the ATP7A gene is located on the X chromosome (Xq13.3), and it is an x-linked recessive disease, consequently affecting mainly male patients. ATP7A gene mutations leading to OHS were mostly splice-site and missense, and to a lesser extent frameshift, deletion, and deep intronic mutations (existing in a region more than 100 base pairs away from the intron-exon junction). ^3^ Nonsense mutations were not found to be associated with this condition, and this may support the suggestion that the presence of 3-5% of wild-type transcripts may lead the patient to develop OHS rather than the more severe form of ATP7A mutations in classical Menkes disease. ^2^ Unfortunately, genetic analysis was not performed in our patient because of unavailability at our facility. Genetic tests should be performed not only for the patient but also for his close relatives, especially the mother determining her carrier status, which may help to estimate the recurrence rate in future pregnancies. ^9^

Occipital horn syndrome partially shares some manifestations with other diseases included in its differential diagnosis. First, other copper transport diseases, especially Menkes disease, which is considered to be more severe genetic variant of OHS. The main point of distinction is that Menkes disease and ATP7A-related DMN manifest themselves with obvious neurological symptoms; the former leads to severe neurodevelopmental delay emerges at a young age while the latter appears in adulthood with a picture very similar to Charcot-Marie-Tooth disease, conversely, OHS shows mild neurological affection if any. ^9^ Other forms of connective tissue diseases are added to the list of differentials, for example, FBLN5- and LTBP4-related cutis laxa, which share signs of redundant skin, urinary abnormalities such as bladder diverticula, and normal mentality; however, OHS is not associated with early onset emphysema, which is common with cutis laxa conditions. Furthermore, OHS has skin laxity in common with dermatosparaxis-type Ehler-Danlos syndrome (dEDS), but it lacks easy bruising, which is characteristic of dEDS. In addition, multiple congenital exostoses can be confused with OHS, whereas multiple exostoses manifest exclusively with skeletal problems due to the development of bone protrusions, which may turn into osteosarcomas. In addition, it is autosomal dominant and affects both males and females equally. ^3^

The management of OHS is not a straightforward task because of the wide range of systems affected in OHS and the unfamiliarity with the condition; hence, it is predictable that the management needs the cooperation of a multidisciplinary team and to be tailored individually. In fact, most of the trials of medication were performed on patients with Menkes disease, the severe genetic allele of OHS. The main goal is close follow-up and early detection of new presentations and complications and to address them properly. The main areas that should be covered at each follow-up visit are the assessment of the patient for dysautonomia symptoms, including blood pressure measurement to exclude orthostatic hypotension, history of fainting attacks, and dizziness. Furthermore, follow-up visits should include screening for urinary anomalies, and educational and developmental assessments. The team of healthcare professionals may include developmental pediatricians to customize an educational strategy, urosurgeons for dealing with bladder diverticula, and physiotherapists and orthopedists to provide medical care to improve musculoskeletal problems. Within the same frame, medicines can be administered to ease the patient’s life, first, antibiotic prophylaxis for urinary tract infection, and second, droxidopa, which is a synthetic precursor for norepinephrine and dopamine, which is proposed to help with dysautonomia symptoms. In addition, copper histidine can also aid in management, with the condition that it should be started well before the age of 3 years; thus, it is important to screen newborn babies for this condition. Kaler et al. suggested that copper histidine showed some benefit in improving neurodevelopmental outcome at the expense of connective tissue-related signs. This could be due to the difficulty of copper histidine joining the TGN to be integrated with cuproenzymes such as the LOX enzyme. ^2^^,^^9^ In fact, none of the previous management methods were initiated at our facility with the expectation that it would be planned for the baby at the genetic center where we referred the case, and hopefully, it would be effective for our baby as he was diagnosed at a very early age.

In accordance with the information provided above, coping with OHS may be quite challenging without early diagnosis and proper management, and unfortunately, can lead to death. In the same context, Beyens et al., in their review of cases, suggested that the mortality rate was 14.7% and the mean age of demise was as early as 19.5 years old. The main causes of fatality were post-surgery apnea attacks, perforated guts, and complicated cephalohematomas with disseminated intravascular coagulopathy. ^4^

Despite all the available knowledge about the condition, many questions have yet to be answered. For instance, are the severity of the symptoms and the age of their onset related to the type of gene mutation or the level of the wild transcript of the protein? Can we use either of them as predictors of disease progression? We hope that more reports and research will be conducted in the future to resolve these issues.

## Conclusion

Here, we report the 35 ^th^ case of occipital horn syndrome, a rare genetic entity in the medical literature. Subtle early presentations, which usually develop with age, may make diagnosis very challenging in early life. Reporting more cases, in addition to genetic counseling and screening for families with already diagnosed members, may encourage a better understanding of the syndrome, expeditious diagnosis, and prompt management.

## Consent

Written informed consent for the publication of clinical data and images was obtained from the patient’s parents.

## List of contributors

Engy M. Hanna, Neonatal intensive care unit (NICU), King Fahad Medical City, Riyadh, KSA. Engymaher230276@gmail.com

## Data Availability

No data are associated with this article.
