# Supplementary material for: Case Report: A male newborn with occipital horn syndrome
Source: F1000Res. 2024 Oct 30;13:864. Originally published 2024 Aug 1. [Version 2] doi: 10.12688/f1000research.154409.2 (PMC11562896; doi:10.12688/f1000research.154409.2)
Supplement: Peer review history

# Reviewer report: Reviewer response for version 2

**DOI:** 10.5256/f1000research.173933.r336508

**Published:** 2024-11-09

**Authors:** Małgorzata Lenartowicz

**Affiliations:** 1 Jagiellonian University, Kraków, Poland

In the current version of the manuscript, the Authors have considered all my comments and answered my questions. In my opinion, the article can be accepted for indexing.

Are enough details provided of any physical examination and diagnostic tests, treatment given and outcomes?

Partly

Is the case presented with sufficient detail to be useful for other practitioners?

Partly

Is sufficient discussion included of the importance of the findings and their relevance to future understanding of disease processes, diagnosis or treatment?

Yes

Is the background of the case’s history and progression described in sufficient detail?

Yes

Reviewer Expertise:

Metabolic disease, animal models of metabolic disease, copper and iron metabolism

I confirm that I have read this submission and believe that I have an appropriate level of expertise to confirm that it is of an acceptable scientific standard.

# Reviewer report: Reviewer response for version 2

**DOI:** 10.5256/f1000research.173933.r336509

**Published:** 2024-11-07

**Authors:** Marwa Elsayed

**Affiliations:** 1 Banha University, Banha, Egypt

I have no further comments

Are enough details provided of any physical examination and diagnostic tests, treatment given and outcomes?

Partly

Is the case presented with sufficient detail to be useful for other practitioners?

Yes

Is sufficient discussion included of the importance of the findings and their relevance to future understanding of disease processes, diagnosis or treatment?

Yes

Is the background of the case’s history and progression described in sufficient detail?

Yes

Reviewer Expertise:

pediatrics and endocrinology

I confirm that I have read this submission and believe that I have an appropriate level of expertise to confirm that it is of an acceptable scientific standard.

# Reviewer report: Reviewer response for version 1

**DOI:** 10.5256/f1000research.169432.r314318

**Published:** 2024-09-03

**Authors:** Małgorzata Lenartowicz

**Affiliations:** 1 Jagiellonian University, Kraków, Poland

The article describes a case of a patient with suspected occipital horn syndrome (OHS) the case is interesting because OHS is an ultra-rare genetic syndrome and the description of each patient here brings information to help build a better clinical picture of the disease.

 However a lot of clinical symptoms indicated that patient suffer from OHS caused by mutation in *ATP7A* gene but unfortunately it should be supported by genetical diagnosis. It can be much more interesting because as no case of this disease has been described in the patient's family so far and it can be a new mutation arised *de novo*.  

 The following issues that arose during the evaluation of the manuscript should be addressed.

**Introduction**

 I suggest making small change to this part of the text as follows:

 “Mutations in this gene lead to either the formation of a distorted form of transmembrane copper transporting P-type ATPase protein or the cessation of its synthesis. ATPase plays a crucial role in copper homeostasis. Being a part of the Golgi network (TGN), it helps to bring copper inside the Golgi network to be incorporated with copper-dependent enzymes include, lysyl oxidase (LOX), which catalyzes the initial step of collagen synthesis. It also comprises the dopamine-β hydroxylase enzyme, which plays a role in the neurological and autonomic systems and helps form norepinephrine out of dopamine. Furthermore, it includes cytochrome C oxidase, superoxide dismutase, ascorbic acid oxidase, and tyrosinase. However, in cases of increased intracellular copper, this protein is mobilized to the cell membrane to help discard the excess.”

**The case report**

 Patient description should be more complete. Please add the information how the child was fed, and whether there were any problems with food intake eg. vomiting. Did the child have diarrhoea? Did the child urinate normally? What was the child's body temperature?

 Authors state that there was no change in the baby's blood analysis results, which is interesting as neonatal jaundice occurred very early. On which day of the child's life was blood drawn for analysis?

**Discussion**

 Information regarding familial history of the patients “On the other hand, most female members of the patients’ families are generally asymptomatic, apart from the pili torti reported in some cases” should be included in the chapter “Case report” not in Discussion.

Are enough details provided of any physical examination and diagnostic tests, treatment given and outcomes?

Partly

Is the case presented with sufficient detail to be useful for other practitioners?

Partly

Is sufficient discussion included of the importance of the findings and their relevance to future understanding of disease processes, diagnosis or treatment?

Yes

Is the background of the case’s history and progression described in sufficient detail?

Yes

Reviewer Expertise:

Metabolic disease, animal models of metabolic disease, copper and iron metabolism

I confirm that I have read this submission and believe that I have an appropriate level of expertise to confirm that it is of an acceptable scientific standard, however I have significant reservations, as outlined above.

# Response

**Published:** 2024-10-24

**Authors:** Marian Georgeos

**Affiliations:** Neonatal intensive care unit, Arab Contractors Medical Center, Nasr City, Cairo Governorate, Egypt

I appreciate the helpful suggestions. In the latest version, I have addressed all the points mentioned, with the exception of the last suggestion regarding relocating the paragraph mentioned in the discussion section (On the other hand, most of female members of the patients' families are generally asymptomatic, apart from the pili torti reported in some cases). I decided to keep it in the discussion section because under the title of the case report introduction, we only discussed the chemical and genetic basis of the disease, without mentioning anything about the symptoms and signs. These were detailed under the discussion section, so I thought it would be less confusing for the reader to keep it there with the rest of the signs and symptoms.

# Reviewer report: Reviewer response for version 1

**DOI:** 10.5256/f1000research.169432.r314325

**Published:** 2024-08-29

**Authors:** Marwa Elsayed

**Affiliations:** 1 Banha University, Banha, Egypt

The article was good in many aspects as

 - It is a case report about one of the uncommon pediatric disorders Occipital horn syndrome (OHS)  which is rarely seen all over the world.

 - The history was brief but included all the important details  , examination findings were systematic  and investigations including imaging scans were very demonstrative .

 However there are some incomplete details such as :

 - treatment and outcomes are not available as the patient was transferred to specialized center for treatment and follow up .

 - Genetic testing was not done due to unavailability at the  facility

 Overall , the article is very beneficial to me as pediatricians and I think it will be for other pediatricians as it gives us data about one of the rare diseases and how to diagnose and treat

Are enough details provided of any physical examination and diagnostic tests, treatment given and outcomes?

Partly

Is the case presented with sufficient detail to be useful for other practitioners?

Yes

Is sufficient discussion included of the importance of the findings and their relevance to future understanding of disease processes, diagnosis or treatment?

Yes

Is the background of the case’s history and progression described in sufficient detail?

Yes

Reviewer Expertise:

pediatrics and endocrinology

I confirm that I have read this submission and believe that I have an appropriate level of expertise to confirm that it is of an acceptable scientific standard.
